# Treatment pathways of lung cancer patients in the Czech Republic: insights from administrative claims data

**DOI:** 10.1136/bmjresp-2024-002653

**Published:** 2026-01-28

**Authors:** Aleš Tichopád, Gleb Donin, Marian Rybár, Vratislav Sedlák, Martin Rožánek, Karla Mothejlová, Vladimír Koblížek, Pavel Turčáni, Milan Sova, Ladislav Dušek, Zuzana Bielčiková

**Affiliations:** 1 Department of Biomedical Technology, Czech Technical University in Prague, Faculty of Biomedical Engineering, Praha, Czech Republic; 2 Department of Respiratory Medicine, University Hospital Hradec Králové, Hradec Králové, Czech Republic; 3 Pulmonary Department, Charles University, Faculty of Medicine in Hradec Kralove, Hradec Kralove, Czech Republic; 4 Center for Pneumology and Interventional Bronchology, Masaryk Memorial Cancer Institute, Brno, Czech Republic; 5 Department of Respiratory Medicine and Tuberculosis, Brno University Hospital, Brno, Czech Republic; 6 Institute of Health Information and Statistics of the Czech Republic, Praha, Czech Republic; 7 Department of Oncology, General University Hospital in Prague, Praha, Czech Republic

**Keywords:** Lung Cancer, Thoracic Surgery, Bronchoscopy, Compliance, Lung Cancer Chemotherapy, Non-Small Cell Lung Cancer

## Abstract

**Introduction:**

A patient pathway is an evidence-based tool that details the phases of care with the aim of increasing the effectiveness and efficiency of patient care. We describe diagnostic and treatment pathways and related overall survival (OS) of non-small cell lung cancer patients.

**Methods:**

This was a longitudinal, historical descriptive cohort study based on administrative claim data, spanning from 2017 to 2022. The index date was determined by the first bronchoscopy with lung biopsy (BX) followed by histopathological (HP) examination, alongside the presence of the International Classification of Diseases 10th revision diagnosis code C34. Incident patients aged ≥18 without prior malignancy. Pharmacotherapies (PHT), including chemotherapy (PHT_CT), precision therapy (PHT_IOTT), as well as surgery (SX) and radiotherapy (RT), were investigated associated with OS. A presence of multidisciplinary team (MDT) and treatment at a Complex Oncological Center (COC) with high-load experience was considered.

**Results:**

We analysed 5819 patient pathways. Less than half (45.6%) of patients had MDT reported within a median of 20 days. Of the 4417 patients treated, 30% underwent more than one BX, 47.7% received PHT_CT, 25.9% underwent SX, 16.4% underwent RT and 9.08% PHT_IOTT. Early initiation of treatment within 4 weeks from BX was identified in 21% of SX patients, 30% of patients treated with PHT_CT and 23% of RT patients. The centralisation of care in COCs primarily concerned SX and PHT_IOTT, while 33% of patients indicated to PHT_CT were treated elsewhere. The median OS reached approximately 16 months in the overall population, 21 months in the verified treated cohort and 13 months in patients treated with PHT_CT, while it was not reached in patients treated with SX. We observed a positive association between patient prognosis and treatment centralisation in COCs.

**Conclusions:**

This methodology can be implemented as a technical infrastructure to fulfil the organisation and quality evaluation routines in cancer care, largely based on administrative data.

**WHAT IS ALREADY KNOWN ON THIS TOPIC**

- There is limited real-world evidence on the impact of structured patient pathways in oncology, although their optimisation is essential for improving cancer control and care efficiency.

**WHAT THIS STUDY ADDS**

- This study develops a robust and simple framework for transforming administrative health data into actionable pathway models.

**HOW THIS STUDY MIGHT AFFECT RESEARCH, PRACTICE OR POLICY**

- Implementation of this approach can enhance transparency and coordination of cancer care, allowing policy of identification and continuous evaluation of quality indicators and supporting national initiatives for optimising and benchmarking oncology services.

## Introduction

 Lung cancer stands as the most fatal cancer globally,^1^ with approximately 85% of patients classified as non-small cell lung cancer (NSCLC).^2^ Recent comprehensive evaluations of lung cancer survival rates in Europe report a 39% 1 year survival rate and a 13% 5-year survival rate, with significant regional disparities.^3^ In the Czech Republic, the 5-year relative survival rate for treated lung cancer patients has doubled over the past 20 years, from 9.6% to 19.3%.^4^

Early identification of the disease is crucial, as surgical resection of NSCLC at an early stage offers favourable prognoses,^5^ with reported 5-year survival rates of up to 70% for small, localised (stage I) tumours.^6^ However, population-based data on lung cancer reveal that over 65% of new cases in the Czech Republic are diagnosed at clinical stage III and IV, with corresponding 5-year stage-adjusted relative survival ratios below 15% and 5%, respectively.^4^ Similar trends are expected globally.^7 8^

Common pathways to diagnosis include general practitioner-ordered imaging and lung specialist attendance without emergency hospital admission, with significant variations in time to diagnosis based on cancer stage.^9^ Access to skilled practitioners capable of performing bronchoscopy with sufficient tissue yield is a key milestone, particularly given patient reluctance to undergo repeat examinations.^10 11^ In the USA, dedicated interventional pulmonology practices have been shown to reduce wait times from abnormal imaging to treatment initiation in newly diagnosed patients.^12^

The multidisciplinary team (MDT) plays a vital role in early care and is presumed to significantly impact clinical outcomes.^13 14^ However, establishing a randomised controlled trial to investigate the effect of MDT remains challenging. Evidence supports the importance of high-volume oncological surgical care^15^ and the necessity of centralising thoracic surgery.^16^ Centralisation of oncological treatment in comprehensive oncological centres (COCs) is assumed to exert a similarly beneficial effect.

To further inform health policy decisions, understanding the key milestones along the patient journey following a suspicious finding is crucial. Since January 2022, lung cancer screening for high-risk individuals has been implemented in the Czech Republic as a nationwide 5-year pilot study.^17^ This initiative has the potential to significantly alter the distribution of cancer stages diagnosed in individuals suspected of having lung cancer. Large electronic health records and administrative data have proven invaluable for understanding pathways between screened and non-screened populations.^918^,^20^ The present study describes diagnostic and treatment pathways and related overall survival (OS) among non-screened patients with NSCLC.

## Methods

### Study design and data sources

This was a longitudinal, historical descriptive cohort study conducted using real-world administrative data from the insured Czech population. The Czech health system comprises six employee insurance funds, each providing nationwide coverage, with all citizens being obligatorily registered with one of them. We used anonymised health administrative claim data from National Registry of Reimbursed Health Services (NRRHS), covering the time period from 2017 to 2022, which provided de-identified information about patient interactions with the healthcare system, including age, sex, medical specialty, International Classification of Diseases 10th revision (ICD-10) diagnoses, and reported date of death. Information on tumour stage was missing from this analysis due to the lack of access to the National Cancer Registry (NCR) database. Patients and/or the public were not involved in the design, conduct, reporting or dissemination plans of this research.

### Study period, index date and cohort selection

**Figure 1:**
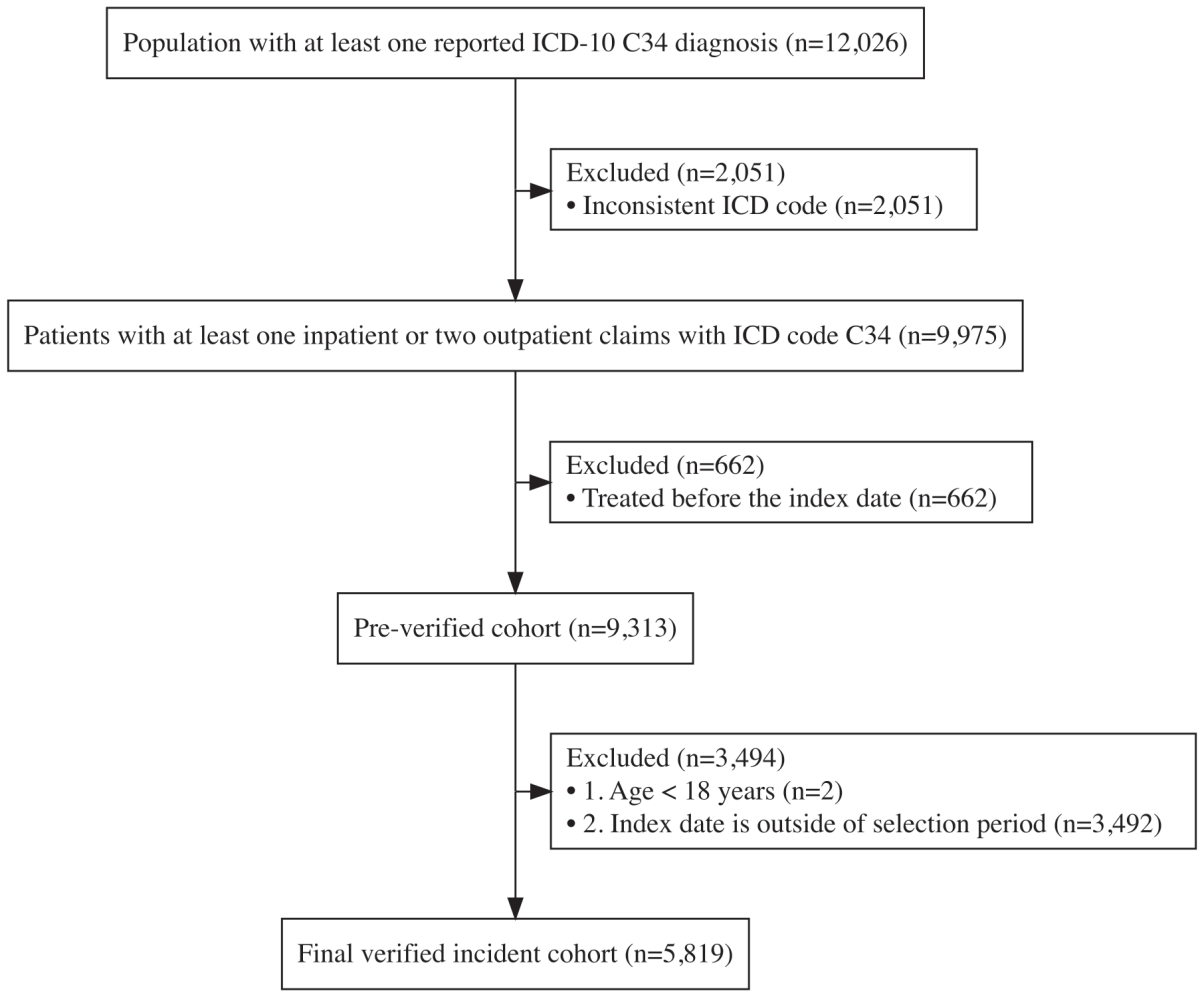
Overview of study design and patient cohort selection. ICD-10, International Classification of Diseases, 10th revision; C34, lung cancer.

The study design and cohort selection are shown in figure 1. The overall study period extended from 1 January 2017 to 31 December 2022, overlapping with the available administrative data period for our study. The study cohort selection period ran from 1 January 2018 until 31 December 2021. Data from 2017 were used to apply exclusion criteria related to verified incidence, ensuring patients had no lung cancer-related records during that year. Patients with less than 12 months of administrative data coverage were excluded (ie, index date after 31 December 2021) to allow adequate follow-up for outcome assessment.

The index date was defined as the date of the first bronchoscopy with lung biopsy (BX) followed by histopathological examination (HP) within the next 60 days, both occurring during the study period. Both standard needle aspiration and endobronchial ultrasound-guided techniques were considered; however, percutaneous biopsy of the lung or pleura was not included among the procedures under investigation. Additionally, the ICD-10 diagnosis code C34 had to be reported on at least one claim document for either the BX or the HP, or on another document of any type within 180 days following the later of these two procedures. The cohort selection was defined as those with an index date falling within the period from 1 January 2018 to 31 December 2021, and aged ≥18 years on the index date. Furthermore, patients were excluded if they had no single inpatient or outpatient claim of any type during 12 months preceding the index date, or if they had a claim related to cancer surgery (SX), radiotherapy (RT) or pharmacotherapy (PHT) prior to the index date.

The patient pathways were presented with process map visualising the traces of patients from diagnostics to death or right censor. Additional process map was presented only for patients who received first treatment, that is, treated patients.

### Pharmacotherapies

Among patients treated with PHT, in addition to the chemotherapy subgroup (PHT_CT), we further subdivided the precision therapy (PHT_IOTT) category. This included anatomical therapeutic chemical (ATC) classification codes for both immunotherapy (PHT_IO) and targeted therapy (PHT_TT). Neoadjuvant chemotherapy (PHT_CT_NEO) was defined as any chemotherapy administered prior to SX and followed by SX within 6 months of its initiation. Because the analysis was limited to the period from diagnosis to treatment and lacked information on disease stage, PHT_CT not followed by SX was most likely considered palliative.

### Follow-up period

The follow-up period was defined as the time from the index date to the first occurrence of any of the following events: (i) the end of the calendar year with the last patient record in administrative data; (ii) the end of study period (31 December 2022); and (iii) death.

### Objectives and outcomes

The objective of the study was to describe the patient pathways to therapy for lung cancer patients in the Czech Republic and their OS during the period before the introduction of preventive screening for lung cancer, that is, prior to 2022.

The Czech Republic’s participation in the Innovative Partnership for Action Against Cancer Joint Action (iPAAC) focuses on the development of a comprehensive Information and Communication Technology (ICT) model.^21^ As part of this effort, we are testing the functionality of our model with the aim of integrating multiple data sources into a national cancer care information system for the collection and publication of cancer care performance indicators.

### Statistical analysis

The statistical analysis was conducted using R for Windows.^22^ In addition to reporting absolute and relative frequencies and employing descriptive statistics for quantitative variables, we conducted a Kaplan-Meier analysis and estimated a log-rank as well as Cox proportional hazards model to study the centralised first-line treatment in COC and the involvement of an MDT review as factors associated with OS. These associations were studied separately for key treatment modalities such as SX, PHT or RT.

## RESULTS

### Study cohort

The overall study cohort consisted of 5819 patients (figure 1), comprising 60% males (3504) and 40% females (2315). The mean age of the cohort at the index was 68 years (SD 9 years). Patients were evenly distributed across the 4 years: 20% (1191) in 2018, 28% (1624) in 2019, 25% (1452) in 2020 and 27% (1552) in 2021. All patients in the study cohort initially underwent lung or bronchial BX, with the majority (69%) having only a single procedure.

The study cohort of treated patients comprised 4.417 patients (details are displayed in online supplemental table S1). Among those, 70% (3092) underwent one BX and 30% (1325) underwent more than one. The median age and sex distribution were similar to those of the total patient population.

### Patient pathways

**Figure 2:**
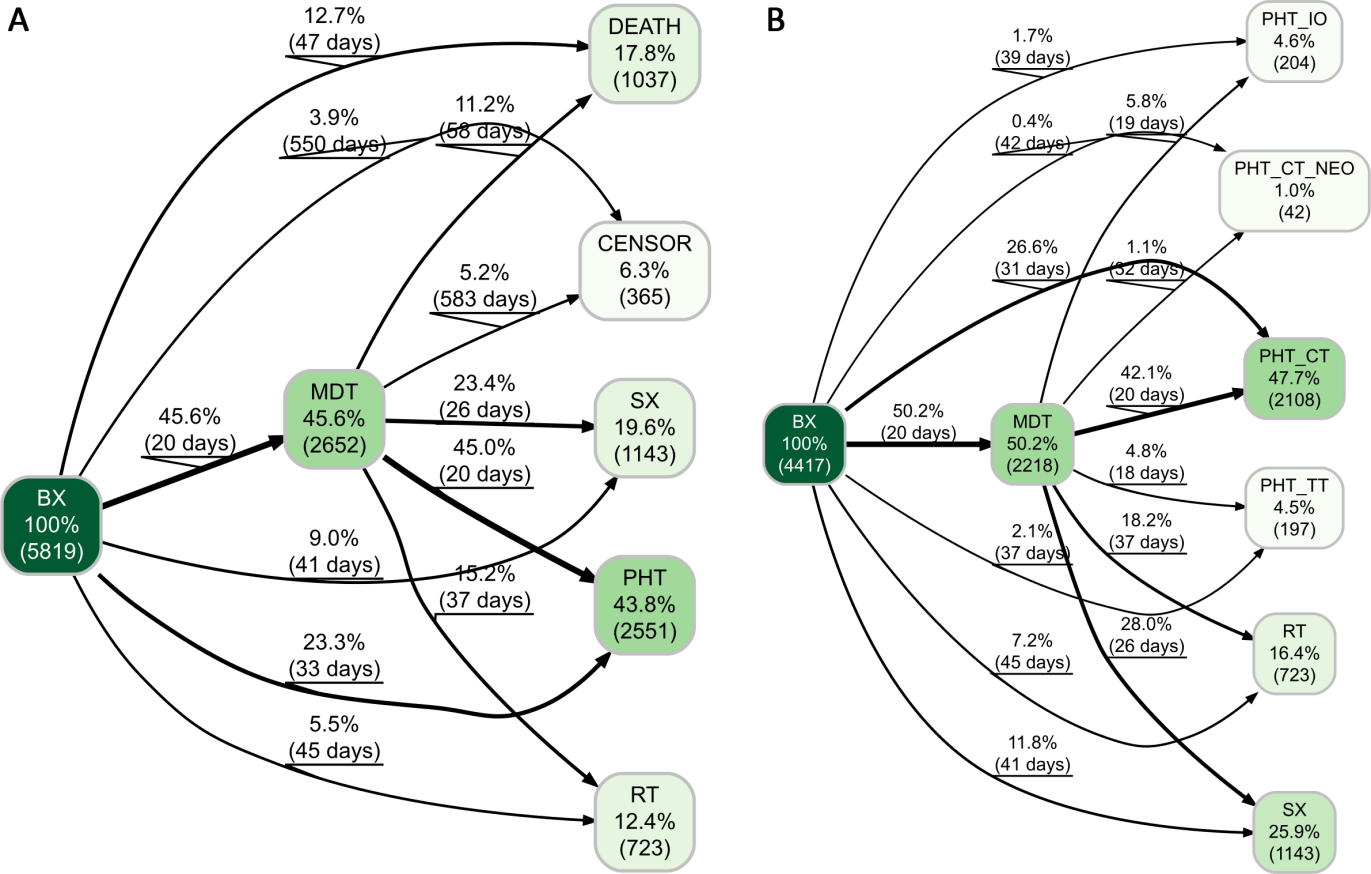
Patient pathway trajectories directly following the initial bronchoscopy in lung cancer patients. (A) The pathways illustrate the progression of overall patients through various stages of the healthcare system after the initial bronchoscopy with biopsy (BX). The numbers in the nodes correspond to the relative and absolute number of patients. The percentage on the edges represents the proportion of the population from the previous node. The number of days on the edges corresponds to the median difference between event onsets. (B) This demonstrates a filtered cohort of patients who receive first treatment after BX. CENSOR indicates no observed event during available follow-up. BX, bronchoscopy; CT, chemotherapy; CT_NEO, neoadjuvant chemotherapy; IO immunotherapy; MDT multidisciplinary team; PHT pharmacotherapy; RT radiotherapy; SX surgery; TT targeted therapy.

Patient pathways are described in figure 2. Following BX, 12.7% (739) of 5819 patients died within a median of 47 days without receiving treatment or undergoing evaluation by the MDT (figure 2A). In total, 6.3% (365) of patients were censored before receiving any treatment, indicating they were lost to follow-up or the study ended before their death could be recorded. Nearly half, 45.6% (2652) of patients received MDT review within a median of 20 days, of whom 11.2% (298) died within 58 days without treatment. Conversely, 23.4% (620) of patients underwent SX with a median time of 26 days, 45.0% (1193) received PTH with a median time of 20 days and 15.2% (403) underwent RT with a median time of 37 days post-MDT.

Among the 4417 treated patients, the distribution of treatments was as follows: 25.9% (1143) of patients received SX, 57.8% (2551) PHT and 16.4% (723) RT (figure 2B). Almost half of operated patients, 45.6% (521), and 55.7% (1174) of patients indicated for PHT were not discussed in the MDT (figure 2B). The expanded PHT options are shown in online supplemental table S1.

### Time to treatment

The median time to treatment initiation in the overall treated population was 43 days (range 27–70) (see online supplemental table S1). Patients treated with PHT_CT had the shortest median at 38 days (range 23–60), possibly due to less demanding diagnostics processes, followed by PHT_CT_NEO at 43 days (range 32–70), and PHT_IOTT at 45 days (range 30–63 days). SX and RT recorded the longest median time to treatment initiation at 47 (range 30–70) and 60 days (range 30–98 days), respectively. Times to treatment associated with specific segments of patient pathways are depicted in figure 2.

We calculated the percentage of patients who initiated treatment within the 4-week, 6-week and 8-week time windows (table 1). In summary, fewer than 25% (calculated as median of numbers in table 1) of patients started treatment within 4 weeks, about 40% within 6 weeks, and approximately 60% within 8 weeks (see also online supplemental figure S1).

**Table 1:** Percentage of patients treated within 4-week, 6-week and 8-week time windows

	SX	RT	PHT
<4 weeks	20.9%	23.1%	30.8%
<6 weeks	41.6%	35.5%	52.8%
<8 weeks	60.9%	47.2%	69.9%

PHT, pharmacotherapy; RT, radiotherapy; SX, surgery.

### Treatment within COC

The majority, 79% (3475 patients), of all treated patients received their treatment within COCs (see online supplemental table S1). The distribution of treatment within COC varied across treatment modalities; practically all, 99% (398) patients who received PHT_IOTT had their treatment in COC, followed closely by 92% (1051) of patients treated with SX. Conversely, only 67% (1,414) of patients receiving PTH_CT were administered within COCs.

**Figure 3:**
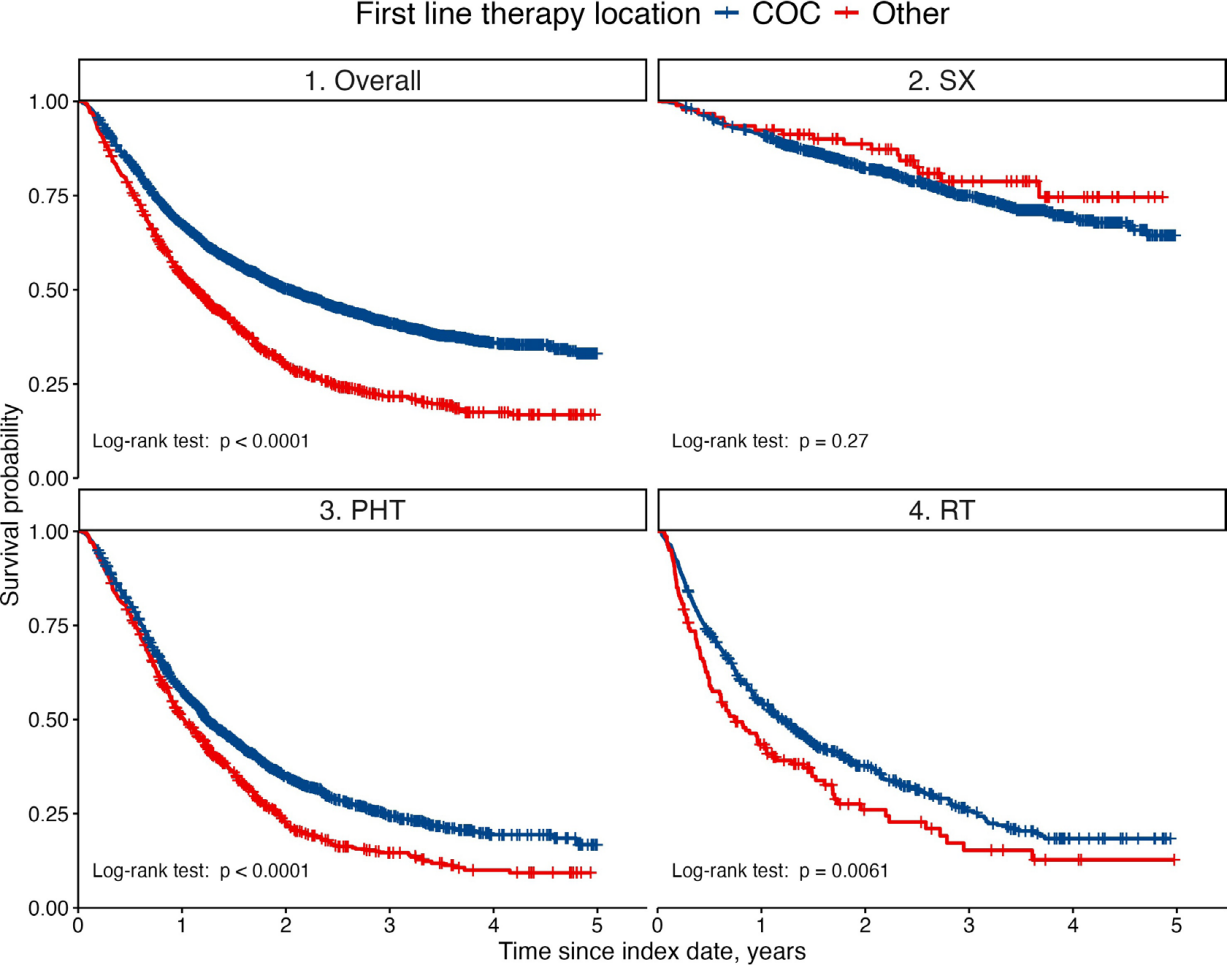
Comparative survival analysis of lung cancer patients treated within Complex Oncological Centers (COCs) or elsewhere, stratified by treatment modality. A Kaplan-Meier estimation with log-rank test (unadjusted) compares patients receiving first treatment within complex oncology centres (COCs) (blue line) versus outside complex oncology centres (Other) (red line). Patients were treated with pharmacotherapy (PHT), including chemotherapy and precision therapy, surgery (SX) or radiotherapy (RT).

We observed a significant difference in OS (Log-rank p<0.001) between patients treated within COC and those treated elsewhere, with the former group seemed to be associated with better OS outcomes over time (figure 3). This holds for patients receiving PHT_CT (Log-rank p<0.0001) and RT (Log-rank p=0.006). For patients undergoing SX, the difference in OS was not significant (Log-rank p=0.27). The results may be biased due to the preferential inclusion of patients with better prognostic profiles treated in COCs.

### Patient outcomes

**Figure 4:**
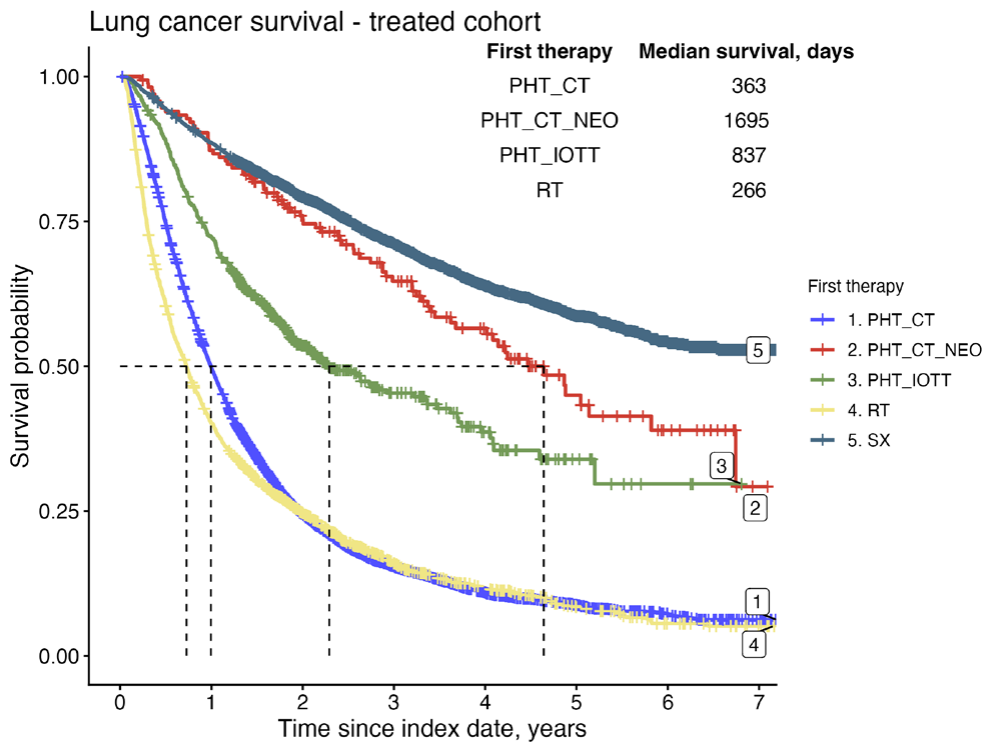
Comparative survival analysis of lung cancer patients by first treatment modality. Survival using a Kaplan-Meier estimation was analysed from the initial bronchoscopy with biopsy and stratified by the first treatment, including chemotherapy (PHT_CT), neoadjuvant chemotherapy (PHT_CT_NEO), precision therapy (PHT_IOTT) comprising immunotherapy (IO) and targeted therapy (TT)*,* radiotherapy (RT)*,* or surgery (SX).

A significant proportion of the overall cohort, 61% (3,557), had died by the end of the study period, with median OS of 485 days (≈16 months). Among those treated, the 2-year OS rate was 46% overall, 83% in patients treated with SX, 76% with PHT_CT_NEO and 27% with PHT_CT (see online supplemental table S2). The median OS reached 633 days (≈21 months) in the verified treated population, 697 days (≈23 months) in patients with advanced disease treated with PHT_IOTT and 385 days (≈13 months) in those treated with PHT_CT. Median OS was not reached in the group of patients treated with SX (figure 4).

Examining the OS outcomes across various treatment pathways for lung cancer patients, trajectories involving SX and PHT_CT_NEO interventions exhibit notably favourable outcomes (see online supplemental figure S2). In general, trajectories with MDT demonstrate enhanced 2-year OS rates, typically ranging from a 7 to 14 percentage point increase over non-MDT pathways (see online supplemental table S3).

## DISCUSSION

The period of the diagnostics as well as immediately following the identification of a lung cancer offers a critical window for effective intervention into the system offering the medical care. Our aim was to provide a clear depiction of the current state in the pathways of lung cancer patients, facilitating a foundation for potential improvements and assessing the impact of upcoming lung cancer screening. We developed a methodology for administrative data processing which can be implemented as a technical infrastructure to fulfil the organisation and quality evaluation of cancer care in the Czech Republic. The comprehensive ICT model developed by Institute of Health Information and Statistics of the Czech Republic, integrating multiple data sources, has been prepared as a legal basis for the collection and publication of cancer care performance indicators. Patient pathway mapping is one of the key quality indicators that need to be implemented near the Czech National Cancer Registry (cancer notification, pathology result) to use administrative reimbursement data in the integrated system for the classification of cancer care.

In our work, we have described the individual points in the patient pathway that can be measured separately in different regions of the Czech Republic and/or at different time points, becoming effective keys for assessing access to care. The study was not designed with confirmatory intentions, nor was it aimed at establishing causality. This is primarily due to the absence of detailed staging information within the dataset, which inherently limits our ability to conclusively rule out selection bias or other confounding factors as explanations for the observed discrepancies. This is why we particularly discuss the importance of analysing the patient pathway as a whole.

An optimal care pathway for lung cancer is still in its preliminary stages across broader health systems.^23^ Significant variations exist in definitions of the intervals used to describe the timeliness of care for lung cancer.^24^ Furthermore, there is little evidence about the effects of pathways used in oncological care.^25^ Conversely, large electronic health records and administrative data have proven invaluable for understanding this subject. Integration of specific quality improvement measures is recommended by the European Respiratory Society in lung cancer care,^26^ and the general objective of the iPAAC^21^ is to develop innovative approaches to advances in cancer control. Pilot 7 evaluates the feasibility of linking population-based cancer registry datasets with administrative and health data sources to describe the complete pathway of cancer patients and to assess the adherence of administered treatments to standard clinical guidelines.

With respect to the missing patient selection, in the discussion of patient trajectories, we will further focus only on time to treatment, access to COCs and involvement of MDTs. Generally, decisions made during the critical period of disease diagnostics have the potential to significantly influence overall outcomes. The principle that cancer should be diagnosed swiftly and promptly treated based on accumulated insights and expertise is widely regarded as historic axiom in oncology.^12 27^ Although we do not report details of the duration of diagnosis in different regions of the Czech Republic, we have shown that 30% of patients underwent more than one BX. Lung puncture as an alternative to histological examination via a BX was not considered in this analysis. The pandemic had an impact on the availability of biopsies and the work of pulmonologists. Additionally, many COVID-19 symptoms overlapped with cancer symptoms, which may have led to late detection of lung cancer. As the time period of the analysis coincided significantly with the period of the COVID-19 pandemic, this overlap may have contributed to over-reporting of deaths (12.7%). Therefore, it would be interesting to examine when and why those who did not receive treatment died, specifically. An analysis including the number of biopsies and the duration of diagnosis may serve as an indicator of the quality of centres performing lung cancer diagnosis. Moreover, an index date starting on the date of the first symptom of lung cancer or the first contact of the patient with a doctor who referred them for a BX would be a better index date, possibly demonstrating the diagnostic delay from the first symptom to the first BX.

Despite repeated attempts to determine the critical time for treatment initiation in lung cancer patients, consistent evidence is lacking,^28 29^ primarily due to methodological heterogeneity in defining the starting point for timing the intervention. A systematic review of European studies examined times to diagnosis and treatment of lung cancer and concluded that they are often longer than recommended.^28^ Our data are consistent with those from Europe, with the median time to treatment ranging between 27–70 days in the Czech Republic, compared with the European Union range of 30–84 days,^28^ which often exceeded published recommendations. Factors associated with timeliness have been incompletely examined, and it remains unclear whether more timely care improves outcomes. Our analysis provides a detailed overview of the time intervals between individual points in the patient’s journey.

We have shown that PHT_CT is associated with the shortest time to treatment initiation (38 days), while the duration for initiating PHT_IOTT is 1 week longer, with a median of 45 days. This reduction could be due to the omission of extensive diagnostic procedures necessary for determining subsequent precision-based therapies, whether administered directly or after the first treatment. Furthermore, preoperative examinations or precise mapping of the affected area for RT are omitted from the process. It is possible that many of those with PHT_CT as the first treatment are prognostically worse patients excluded from indication for PHT_IOTT due to poorer performance status. Time to PHT_IOTT is critical for patients in the advanced stage of lung cancer and could be potentially measured as quality indicator in COCs.

A recent study suggests that a time to SX longer than 4 weeks is associated with an increased rate of recurrence and death.^30^ One of the reasons for the ambiguity in this area is the precise determination of the moment when the patient should be urgently directed towards further steps. There is an apparent paradox, where highly suspicious patients with well-manifested diseases will be promptly managed and referred for targeted examinations before treatment or directly to treatment. However, these patients will likely suffer from advanced stages of the disease, and the benefit of early intervention will likely result in only a mild extension of life. Conversely, for patients in early stages of the disease (stages I and II), where early precise determination and subsequent treatment can lead to healing, the duration to treatment is longer due to uncertainty, new and repeated examinations, which apparently have a prognostically unfavourable impact. Czech data presented by our analysis are unsatisfactory because they show that only 21% of lung cancer patients undergo SX within 4 weeks, and even within 8 weeks from diagnosis, the number of operated patients does not exceed 70%. However, the observed period includes the time before (2018–2019) and after (2020–2021) the centralisation of surgical treatment in the Czech Republic, which may have an impact on the duration to SX. Inclusion of disease stage is crucial for accurate monitoring of this indicator.

Additionally, it is plausible that treatments, particularly those like PHT_CT that are more immediate and less reliant on extensive diagnostics, are commenced directly at the centre where the diagnosis was made, avoiding patient delegation to COCs. Centralised care in high-volume centres ensures that patients benefit from the expertise and resources available in such settings, potentially leading to more accurate diagnoses and tailored treatment plans. Despite challenging methodology, emerging evidence suggests that centralised high-volume care, the involvement of MDT and minimising time to treatment initiation are pivotal factors that could enhance OS rates for lung cancer patients.^15 16 28 30 31^ The most complex treatment is provided in a network of 15 COCs in the Czech Republic, 10 of which are certified lung cancer centres and seven are highly specialised thoracic surgery cancer centres.^4^ We have shown that lung cancer SX and personalised treatment are predominantly centralised in our country which is good starting point for improving the quality of care. Our comparison of the prognosis of patients treated with SX in COC versus elsewhere was limited by the fact that 92% of patients fell into the first group, so it can be biased. Although the OS data of operated patients are in line with those published,^10^ interpreting them without staging data must be done with caution. Conversely, patients treated with PHT and RT significantly benefited from centralised treatment. A possible explanation for the observed better survival among patients receiving chemotherapy within COCs includes both causal and selection mechanisms. First, a true causal effect may exist: high-volume comprehensive centres offer better access to multidisciplinary expertise, advanced diagnostics and complex supportive care, which may translate into improved treatment tolerance and outcomes.^13^,^16^ Second, a selection effect is also likely to play a role. Patients with very poor performance status or rapidly progressing disease may never reach a COC and instead initiate treatment locally, which could artificially inflate survival differences. Distinguishing between these mechanisms would require randomised allocation or at least detailed staging and clinical covariates, which were not available in our administrative dataset. Nevertheless, both mechanisms merit further investigation, as they have important implications for centralisation policy and resource planning. However, the challenge remains to ensure early access to both SX and PHT care for lung cancer patients across the regions of the Czech Republic.

Most studies focused on the association between MDT and outcomes are, among other reasons, non-randomised and non-blinded for ethical considerations. The existing evidence does not unequivocally indicate an association between MDT and OS in lung cancer patients.^13 14^ We showed a trend of better OS in trajectories involving an MDT review; however, caution must be exercised without considering the underlying patient selection processes. For example, patients with better performance status may be preferentially discussed in MDT meetings. It is particularly important that almost half of SX patients do not undergo an MDT review. It is possible that in some patients who underwent MDT, this consultation was not documented. Although the reporting of the signal code for MDT, as we know it now, has been in effect since 1 January 2017, our data show that the reporting of MDT has been increasing over time. It is also worth mentioning that the data collection system for insurance companies, known as care reporting, is not standardised in the Czech Republic.

Screening of high-risk individuals, as initiated in the Czech Republic and elsewhere, may deliver load of early-stage patients with potentially curable stages.^17 32^ While historical incidence rates for pulmonary nodules in the USA remained steady for many decades at 150 000 per year,^33^ a more recent evaluation using electronic health records and natural language processing increased the incidence 10-fold to over 1.5 million nodules detected per year.^34^ This will be a substantial game-changer. The opportunity window for curable SX in those early diagnoses demands a prompt and timely response to be effectively used. Conversely, the influx of new patients may quickly create a capacity bottleneck in the centres. Mapping the patient journey is very practical in this regard, highlighting the system’s limits in ensuring timely access to treatment or, conversely, in measuring the effectiveness of care centralisation. Measuring time to treatment over time can serve as a quality indicator for benchmarking care-providing centres.

As discussed above, our analysis has several limitations: the absence of disease stage and histopathological subtype; the lack of analysis of patients diagnosed by CT-guided biopsy and biomarker testing; the absence of data on performance status and causes of death; the use of the biopsy date as the index date rather than reflecting the onset of disease symptoms; and the absence of standardised reporting of care in the Czech Republic. We also noted possible effects from the COVID-19 pandemic during the study period. Furthermore, our analysis was limited to administrative data from five of the six payers. However, we consider the results of patient trajectories as secondary in this case. Considering the aim of the study, we believe that the main contribution of the analysis lies in the concept of trajectories as a tool for mapping the quality of care itself.

## CONCLUSIONS

Mapping the patient pathway serves as a quality indicator. Our analysis showed that 79% (3475) of patients receiving first treatment were treated in COCs, 50.2% (2218) underwent MDT review, and the median time to treatment initiation was 43 days with only approximately 25% of treated patients initiated treatment within 4 weeks. The emphasis on these factors extends beyond clinical considerations to encompass economics and logistics, underscoring the importance of efficient healthcare delivery systems that optimise patient outcomes while considering cost-effectiveness. This evidence provides the foundation for developing guidelines and policies aimed at optimising care delivery in high-volume centres (COCs in the Czech Republic), where such interventions are most feasible and likely to yield significant benefits for patients. As the healthcare community continues to strive for improved lung cancer OS rates, these factors should remain at the forefront of early intervention strategies, highlighting the need for ongoing research and evidence-based practice in this evolving field.

## Supplementary material

10.1136/bmjresp-2024-002653 online supplemental file 1

## Data Availability

Data may be obtained from a third party and are not publicly available.
