# Supplementary material for: Treatment pathways of lung cancer patients in the Czech Republic: insights from administrative claims data
Source: BMJ Open Respir Res. 2026 Jan 28;13(1):e002653. doi: 10.1136/bmjresp-2024-002653 (PMC12853478; doi:10.1136/bmjresp-2024-002653)
Supplement: online supplemental file 1 [file bmjresp-13-1-s001.pdf]

Supplementary material

Table S1 Characteristics of patients treated for lung cancer diagnosed from January 2018 through 2021

	Overall<br>N = 4,417	CT<br>N = 2,108	CT_NEO[3]<br>N = 42	IOTT<br>N = 401	RT<br>N = 723	SX<br>N = 1,143
**Age**	68 (63, 73)	68 (63, 73)	66 (62, 70)	67 (60, 73)	71 (66, 76)	67 (62, 72)
**Males**	2,605 (59%)	1,297 (62%)	23 (55%)	214 (53%)	449 (62%)	622 (54%)
**Females**	1,812 (41%)	811 (38%)	19 (45%)	187 (47%)	274 (38%)	521 (46%)
**Survived[1]**	1,897 (43%)	561 (27%)	31 (74%)	195 (49%)	234 (32%)	876 (77%)
**Died[1]**	2,520 (57%)	1,547 (73%)	11 (26%)	206 (51%)	489 (68%)	267 (23%)
**Treatment detail**						
**Any PHT**	87 (2.0%)	78 (3.7%)	9 (21%)	0 (0%)	0 (0%)	0 (0%)
**CT**	2,063 (47%)	2,030 (96%)	33 (79%)	0 (0%)	0 (0%)	0 (0%)
**IO**	204 (4.6%)	0 (0%)	0 (0%)	204 (51%)	0 (0%)	0 (0%)
**TT**	197 (4.5%)	0 (0%)	0 (0%)	197 (49%)	0 (0%)	0 (0%)
**Only one BX**	3,092 (70%)	1,474 (70%)	25 (60%)	243 (61%)	485 (67%)	865 (76%)
**More than one BX**	1,325 (30%)	634 (30%)	17 (40%)	158 (39%)	238 (33%)	278 (24%)
**MDT absent[2]**	2,199 (50%)	1,174 (56%)	17 (40%)	167 (42%)	320 (44%)	521 (46%)
**MDT reported[2]**	2,218 (50%)	934 (44%)	25 (60%)	234 (58%)	403 (56%)	622 (54%)
**Treatment in COC**	3,475 (79%)	1,414 (67%)	29 (69%)	398 (99%)	583 (81%)	1,051 (92%)
**Patients with advanced disease treated with PHT_IOTT treatment not in COC**	942 (21%)	694 (33%)	13 (31%)	3 (0.7%)	140 (19%)	92 (8.0%)
**Median time to treatment**	43 (27, 70)	38 (23, 60)	43 (32, 70)	45 (30, 63)	60 (30, 98)	47 (30, 70)
**Time to treatment grouped**						
**[0,30)**	1,360 (31%)	796 (38%)	7 (17%)	93 (23%)	180 (25%)	284 (25%)
**[30,60)**	1,636 (37%)	778 (37%)	21 (50%)	192 (48%)	180 (25%)	465 (41%)
**[60,120)**	1,095 (25%)	434 (21%)	14 (33%)	90 (22%)	240 (33%)	317 (28%)
**[120+)**	326 (7.4%)	100 (4.7%)	0 (0%)	26 (6.5%)	123 (17%)	77 (6.7%)

The verified treated cohort comprised of 4.417 patients in median age of 68 years.
[1]Survival analysis covers the period 2018-2021. [2]Some MDT consultations could have been reported under different codes, such as individual claims for each healthcare provider specialist, or they might not have been reported at all. [3]CT prior SX, following no later than 6 month after its onset.
PHT, pharmacotherapy; CT, chemotherapy; CT_NEO, neoadjuvant chemotherapy; IO, immunotherapy; TT, targeted therapy; BX, bronchoscopy; RT, radiotherapy; SX, surgery; MDT, multidisciplinary team; COC, complex oncological center.

Figure S1 Cumulative distribution of first treatment since the initial bronchoscopy with biopsy, and time to treatment periods

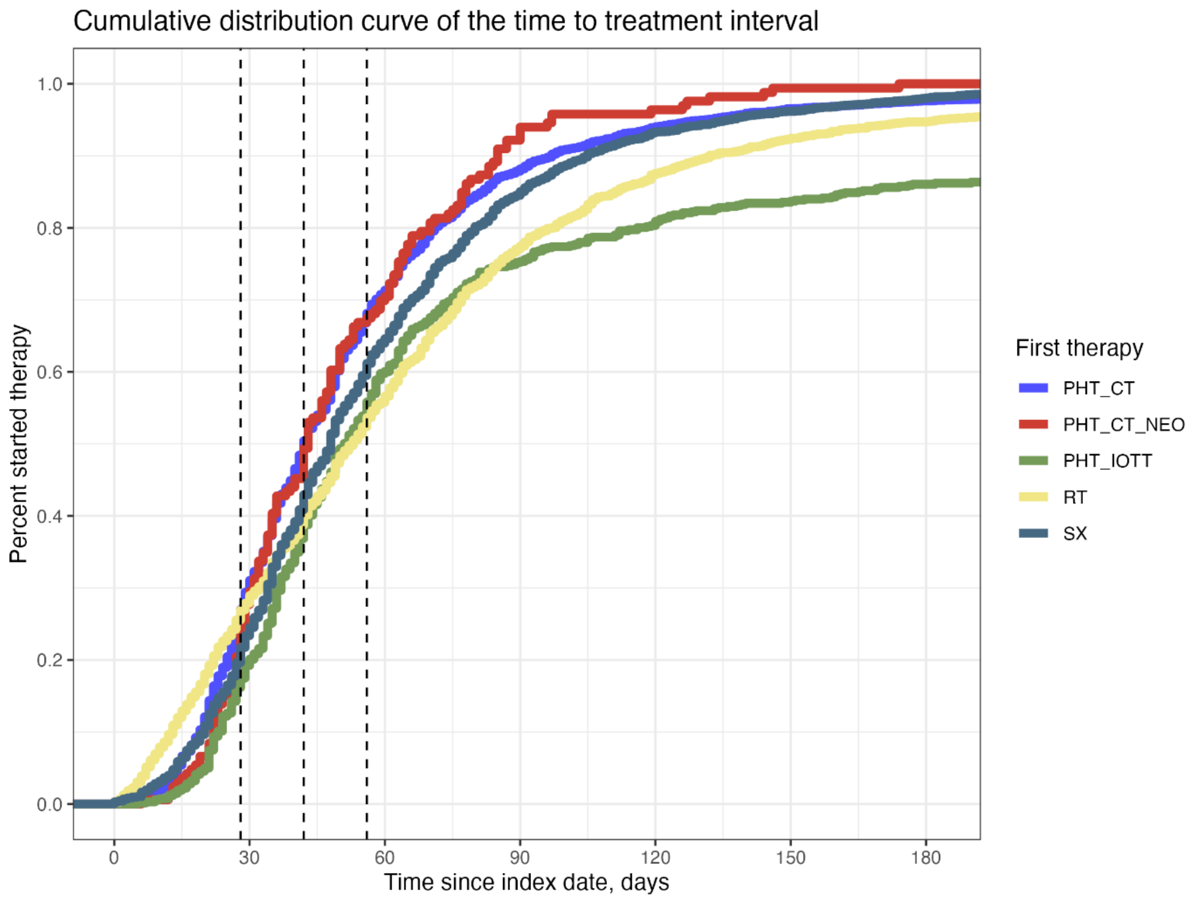

Analysis contains data of treated patients receiving treatment as follows: chemotherapy (PHT_CT), neoadjuvant chemotherapy (PHT_CT_NEO), precision therapy comprising immunotherapy and targeted therapy (PHT_IOTT), radiotherapy (RT), or surgery (SX) since the initial bronchoscopy. Dashed lines indicate the four, six and eight-week interval which indicate the differences among time to treatment.

Figure S2 Comparative survival analysis of lung cancer patients by pathway trajectory

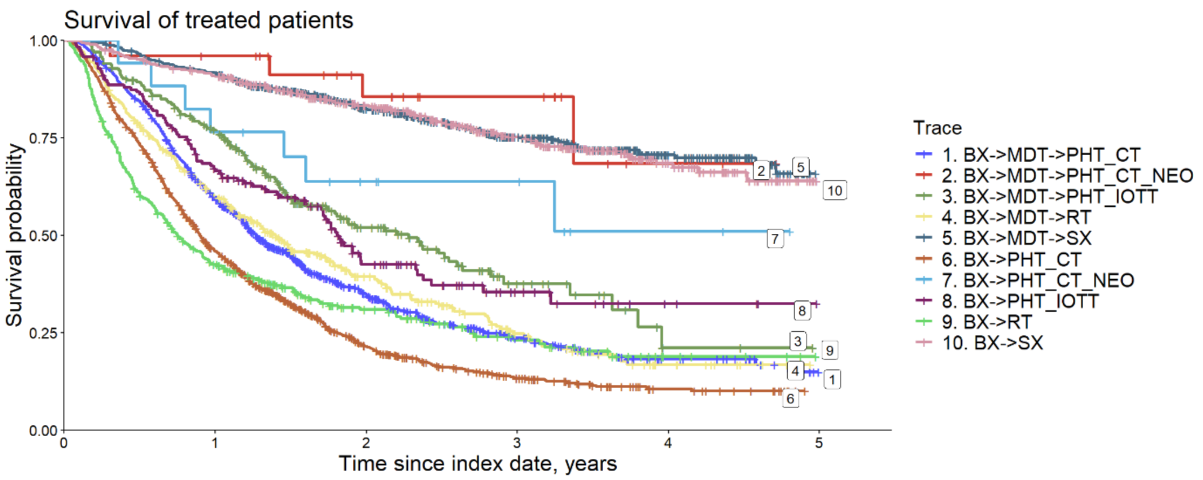

Survival using a Kaplan-Meier estimation was analyzed since the initial bronchoscopy with biopsy (BX) and stratified by their trajectory to the first treatment in treated patients; chemotherapy (PHT_CT), neoadjuvant chemotherapy (PHT_CT_NEO), precision therapy comprising immunotherapy and targeted therapy

(PHT_IOTT), radiotherapy (RT), or surgery (SX) either directly or via multidisciplinary team review (MDT). Trajectories involving SX and PHT_CT_NEO interventions exhibit notably favorable.

Table S2 Survival outcomes according to first line treatment from the index date

First treatment	Median survival (Days)	Median Survival 95% CI	SR 90 Days	SR 180 Days	SR 365 Days	2-Year SR
All patients	633	596 - 665	92%	82%	64%	46%
PHT_CT	385	363 - 415	91%	78%	52%	27%
RT	400	349 - 476	86%	70%	52%	36%
SX	NA	NA - NA	99%	96%	91%	83%
PHT_IOTT	697	639 - 866	94%	88%	73%	48%
PHT_CT_NEO	NA	1231 - NA	100%	95%	88%	76%

CI, confidence interval; PHT_CT, chemotherapy; PHT_CT_NEO, neoadjuvant chemotherapy; PHT_IOTT, precision therapy; RT, radiotherapy; SR, survial rate; SX, surgery.
The 95% confidence intervals (CI), and survival rates (SR) at various time points post-treatment initiation are provided. "NA" indicates data not available or not applicable. Median OS of operated patients was nor reached.

Table S3 Survival outcomes by patient pathway trajectory since index date

Trajectory	Median survival (Days)	Median Survival 95% CI	SR 90 Days	SR 180 Days	SR 365 Days	2-Year SR
BX	75	63 - 86	46%	35%	28%	21%
BX-MDT	198	150 - 279	66%	51%	41%	28%
BX-SX	NA	NA	98%	95%	91%	83%
BX-MDT-SX	NA	NA	99%	96%	92%	82%
BX-RT	274	239 - 341	80%	60%	43%	31%
BX-MDT-RT	510	434 - 630	91%	78%	60%	39%
BX-PHT	353	329 - 383	89%	75%	49%	25%
BX-MDT-PHT	525	486 - 569	94%	85%	64%	39%
BX->MDT->PHT_CT_NEO	NA	1231 - NA	100%	96%	96%	86%
BX->PHT_CT_NEO	NA	584 - NA	100%	94%	76%	64%
BX->PHT_CT	329	313 - 351	88%	73%	46%	22%
BX->MDT->PHT_CT	463	436 - 511	94%	84%	59%	35%
BX->PHT_IOTT	660	622 - 854	92%	86%	67%	42%
BX->MDT->PHT_IOTT	833	639 - 963	96%	89%	77%	52%

The pathway trajectories are categorized based on the involvement of bronchoscopy with biopsy (BX), multidisciplinary team consultations (MDT), pharmacotherapy (PHT), radiotherapy (RT), and surgical interventions (SX). Among PHT, chemotherapy (PHT_CT), precision therapy (PHT_IOTT), and neoadjuvant chemotherapy (PHT_CT_NEO) were further defined. The 95% confidence intervals (CI), and survival rates (SR) at various time points post-treatment initiation are provided. "NA" indicates data not available or not applicable. Median OS of operated patients was nor reached. The index date was operationalized as the date of the first BX. In general, trajectories with MDT demonstrate enhanced OS rates compared to pathways without MDT review.
